# Frequency of Dental Caries in Four Historical Populations from the Chalcolithic to the Middle Ages

**DOI:** 10.1155/2011/519691

**Published:** 2011-11-22

**Authors:** A.-M. Grimoud, S. Lucas, A. Sevin, P. Georges, O. Passarrius, F. Duranthon

**Affiliations:** ^1^Service d'Odontologie, Hôtel-Dieu Saint Jacques, 2 rue Viguerie, TSA 60035, 31059 Toulouse cedex 9, France; ^2^Laboratoire d'Anthropologie UMR 5288, 37 Allées Jules Guesde, 31073 Toulouse cedex 3, France; ^3^Laboratoire PACEA UMR 5199, CNRS, Avenue des Facultés, 33405 Talence cedex, France; ^4^Pôle d'Archéologie Départemental, Conseil Général des Pyrénées Orientales, 66000 Perpignan, France; ^5^Museum d'Histoire Naturelle, 35 Allées Jules Guesde, 31000 Toulouse, France

## Abstract

The majority of dental carie studies over the course of historical period underline mainly the prevalence evolution, the role of carbohydrates consumption and the impact of access to dietary resources. The purpose of the present investigation was to compare population samples from two archaeological periods the Chacolithic and Middle Age taking into account the geographical and socio economical situation. The study concerned four archaelogical sites in south west France and population samples an inlander for the Chalcolithic Age, an inlander, an costal and urban for the Middle Age. The materials studied included a total of 127 maxillaries, 103 mandibles and 3316 teeth. Data recorded allowed us to display that the Chalcolithic population sample had the lowest carie percentage and the rural inlander population samples of Middle Age the highest; in all cases molars were teeth most often affected. These ones differences could be explained according to time period, carious lesions were usually less recorded in the Chalcolithic Age than the Middle because of a lesser cultivation of cereals like in les Treilles Chacolithic population sample. In the Middle Age population samples, the rural inland sample Marsan showed the highest frequency of caries and ate more cereal than the coastal Vilarnau and the poor urban St Michel population samples, the first one ate fish and Mediterranean vegetal and fruits and the second one met difficulties to food access, in both cases the consumption of carbohydrates was lesser than Marsan population sample who lived in a geographical land convice to cereals cultivation.

## 1. Introduction

Studies of dental caries over historical periods allow us to know and follow the evolution of the frequency of the disease and also its association with environmental resources, especially the relationship, now well established, between formation of caries, type of food consumed, and lifestyle [1–3].

Observations around the aetiology of carious lesions identify the role played by carbohydrates, with sugar [4] as the main factor involved in the increased prevalence of carious lesions, which arises with the cultivation of cereals and the possibility of cooking them, because cooking makes the carbohydrates soft, thus allowing them to stick to teeth, and, moreover, modifies the carbohydrate makeup by cutting the chains into shorter pieces [5, 6]. Overall, these modifications make the carbohydrate more cariogenic. Nevertheless, multiple factors appear to be involved in dental caries, including oral ecosystem compounds and salivary gland function [7]. The Keyes' diagram summarizes the main areas of interactions involved in the dental carious process, that is, hygiene practices, oral environment, quality of food consumed [7, 8], and, above all, the time factor: how many times a day food intake occurs.

Our text is part of the history of carious lesions and the relationship between frequency of caries and quality of food intake [6, 9], which broadly depends on the socio-economic context, including access to food resources and means of cooking. Taking these caries parameters into account, we propose to compare the frequency of caries (i) between two samples of populations who lived during two different historical periods, that is, the Chalcolithic and the Middle Ages, and (ii) among three samples of populations, of which two were rural and one was urban, who lived in different socio-economic and geographical contexts in south-west France in the Middle Ages [10]. For the three medieval samples, we compare frequency of carious lesions, firstly among the two rural population groups, one that lived on the southwest Mediterranean coast and the other that inhabited an inland region, and secondly rural and urban population samples [3, 11].

Given the age of all the collections studied, it is necessary to present the state of preservation of the jaws used to determine the carious lesion frequency.

With this in mind, we chose to verify both of the following hypotheses: firstly, for two different historical periods, a thousand years apart, dental carious lesions are more frequent in population samples living in the Middle Ages than in the Chalcolithic and, secondly, Medieval population groups living in different socio-economic and geographical contexts show differences in caries frequency.

## 2. Materials and Methods

### 2.1. Archaeological Site Contexts and Populations Studied

**Figure 1:**
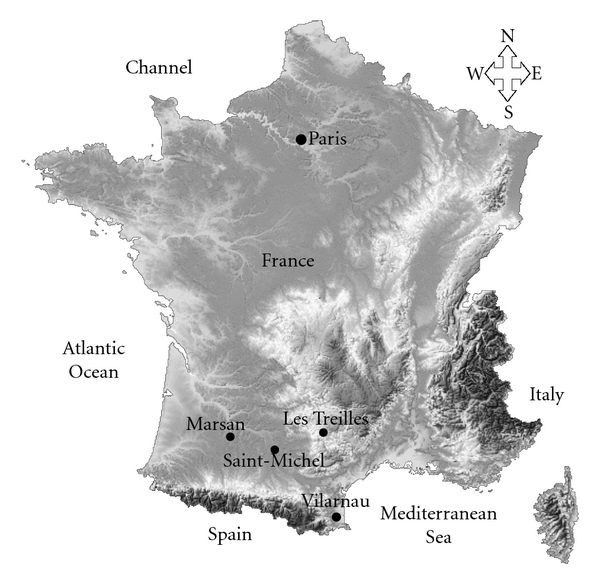
Topographic distribution of the four archaeological sites in southwest France.

The present research was carried out on the skeletal remains of adults selected among the individuals excavated from the following 4 sites in southwest France (Figure 1) [10].

- The Les Treilles cave, a rural inland burial ossuary site dating from the Chalcolithic Age (2 600–1 700 BC), situated in a mountainous region where the soil is not suitable for the cultivation of cereals.
- The Marsan-Lasserre Medieval Cemetery (10th–12th C.), situated at a rural inland site conducive to cereal cultivation. At this period, people used to eat cooked cereal daily, the starch of which formed one of their main food resources.
- The Saint Michel Medieval Urban Cemetery (12th–14th C.) situated in the Toulouse suburb of Saint Michel, which was inhabited by very poor people who had difficulties in obtaining food because of limited access to food resources.
- The Medieval Rural Cemetery of Vilarnau (12th–14th C.) situated near the Mediterranean Coast, a region which enjoys a milder climate than the other three sites studied; this geographical context allowed vines, fruits, and vegetables to be cultivated and gave access to sea foods.

The samples studied in each of these four archaeological sites included (i) 24 mandibles for the Les Treilles Ossuary, (ii) 33 pairs of jaws belonging to 33 individuals in the Marsan collection, (iii) 41 pairs of jaws belonging to 41 individuals at the Saint-Michel Cemetery, and (iv) 58 pairs of jaws belonging to 58 individuals at the Vilarnau Cemetery. This provided a total of 103 maxillae, 127 mandibles, and 3316 teeth (Table 1).

### 2.2. Dental Parameters

Each tooth was recorded with reference to the maxillary state, with presence or absence of teeth and carious lesions.

Data were recorded by the same team using a previously published method [12].

#### 2.2.1. Criteria for Antemortem and Postmortem Tooth Loss

To evaluate the degree of preservation and level of maxillary pathology in the four collections studied, we recorded the missing teeth as being lost antemortem or postmortem, which provided information regarding the condition and percentage of teeth lost because of diseases. When a part of the alveolar bone and the corresponding tooth were absent, we recorded them as undetermined.

Antemortem tooth loss (AMTL) was recorded if there were signs of bone remodelling at the level of the alveolar socket, and Postmortem tooth loss (PMTL) if there was clear evidence of an empty alveolar socket.

#### 2.2.2. Criteria for Selection of Maxillary Samples

In the four archaeological collections, all jaws were selected according to the level of damage as defined by [11], and only those belonging to levels 1 and 3 were studied.

Data were recorded by the same team using the same method as previously published [12].

- Level 1: indicating preservation of both maxilla and mandible and preservation of more than 50% of alveolar bones.
- Level 2: indicating preservation of both maxilla and mandible but with preservation of less than 50% of alveolar bones.
- Level 3: indicating preservation of only the maxilla or the mandible and preservation of more than 50% of alveolar bones.
- Level 4: indicating preservation of only the maxilla or the mandible and preservation of less than 50% of alveolar bones.

Following these levels, only adults were selected for this research. Children were not studied because their skeletons were poorly preserved.

Overall, 103 maxillae and 127 mandibles with at least six teeth on the dental arch were studied; all retained roots were recorded as remaining teeth. A total of 3535 teeth were studied (Table 1).

#### 2.2.3. Criteria for Recording Carious Lesions

To diagnose carious lesions we used simple stages to avoid subjectivity in the scoring and eliminate false diagnosis. Thus, a carious lesion was defined as a clear cavitation in tooth tissue [6] detected macroscopically under the right lighting with the naked eye and using a dental probe in case of doubt regarding lesion development [13].

We differentiated between carious lesions and other tooth surface defects like pits and deep fissures; also colour changes of the enamel were not considered as caries unless there was cavitation underneath [6]. All sticky fissures and early decalcification without loss of enamel were disregarded because they could have introduced an element of doubt [6].

The carious lesions were recorded considering their topographical location according to tooth part, the surfaces affected, and whether or not the pulp chamber was penetrated, as follows:

- coronary location on occlusal, mesial, distal, lingual, buccal surfaces,
- radicular location on mesial, distal, lingual, and buccal surfaces,
- cervical location on mesial, distal, lingual, and buccal surfaces,
- pulpar exposure.

All teeth were examined twice by direct inspection at an interval of two weeks with the same team and method. Only surfaces that were considered to be carious at both examinations were taken into account.

#### 2.2.4. Statistical Method

The data were processed with SPAD software. We analysed results using discriminant analysis, a technique for classifying a set of observations in predefined classes. The purpose is to determine the class of an observation based on a set of variables known as predictors or input variables. The rows of the data matrix to be examined constitute points in a multidimensional space, as do the group mean vectors. Discriminating axes are determined in this space, in such a way that optimal separation of the predefined groups is attained.

## 3. Results and Discussion

The data recorded in Table 1 concern the state of the dental arches for the four population samples from the four archaeological sites of southwest France.

Table 1 shows the number of teeth expected, the numbers and percentages of teeth recorded present and absent, and the number of jaw samples from the four archaeological sites studied. For the Chalcolithic collection, only 24 mandibles were present; the remaining jawbones could not be examined because of their poor state of preservation. The percentage of tooth presence was 40.8%, which was smaller than for Marsan 74.9%, Saint-Michel 75.6%, and Vilarnau 74.1%. The percentage of teeth lost postmortem in Les Treilles was 47.3%, higher than that* in *Marsan 13.2%, Saint-Michel 8.6%, and Vilarnau 15.8%, and the there were not pulpar caries. This poor state of preservation can be explained because the Chalcolithic collection was discovered long ago, in 1939 in an ossuary, and the bones were handled many times at a period when anthropological considerations were different.

This observation led us to consider the limitations of the archaeological samples studied as follows. (i) in general, over time, bones and teeth undergo damage or taphonomic effects related to bad conditions of preservation, occurring, for instance, during long contact with the soil in the osteological series where graves were not individual or had deteriorated over time because of the basic coffin structure. Nevertheless we stress that teeth resist better than bone in extreme conditions of preservation because of their high degree of mineralization. (ii) In our study the limitation concerned (1) the PMTL, which most often affected incisors and premolars, due to the nonretentive shape of their roots as often found, and (2) the better state of preservation of mandibles than maxillae because the maxillary bone structure is more fragile than that of the mandibles. That explains why maxillae were missing in the oldest collection whereas, in the Medieval osteological collection, we were able to select paired maxillae and mandibles although the maxilla was generally in a poorer state than the mandible [14]. Moreover it is difficult to have access to information about food intake, dental hygiene, and previous state of health.

In Table 2, we give the frequency of presence or absence and the distribution of carious lesions according to tooth type: incisor, canine, premolar, and molar in the four population samples studied in Les Treilles, Marsan, Saint-Michel, and Vilarnau.

Overall comparison between sites showed the teeth most affected by (i) antemortem tooth loss (AMTL) were the molars in the four population samples and by (ii) postmortem tooth loss (PMTL) were the anterior teeth because of the nonretentive form of their roots. In both cases, the oldest collection, Les Treilles, was the most affected.

As for the impact of AMTL frequency on caries prevalence, we note that (i) the AMTL frequency is low and so its influence is negligible and (ii) tooth loss can be due to wear level. Indeed, in a study by Lucas et al. [12], tooth wear, the main dental feature in historic populations, is reported to be associated, in case of heavy tooth wear, with periapical lesion development via pulpar necrosis. Thus, the first molars, the most worn teeth, are also the most often absent antemortem.

Considering the distribution of carious lesions, we can see that, overall, the Les Treilles archaeological site was the least and Marsan the most affected for all tooth types. Also, for the four population samples, molars were the most affected tooth type and canines and incisors the least. Regarding the higher degree of severity of caries when root or pulp were affected, the four population samples showed that molars were the most often affected, followed by premolars. Canines and incisors were affected in the three medieval sites: Marsan, Vilarnau, and Saint-Michel.

Regarding the types of carious lesions, Marsan provided the population sample most affected by all types of caries and, moreover, showed the highest levels of severity, that is, pulpar and radicular cavities.

Bearing this in mind, we considered (i) the two historical periods studied, separated by more than 1000 years, and found that the Les Treilles population had the lowest percentage of teeth affected by caries compared to the three medieval populations, a finding consistent with other studies [15], and (ii) the geographical and socio-economic context, including food resources. We explained the difference of caries incidence between the Chalcolithic and Middle Ages by a diet with a lower consumption of carbohydrates and cooked food in the Chalcolitic Age, when cereal cultivation was less common than in the Middle Ages in general and especially for the population of Les Treilles, who lived in a mountainous area not conducive to cereal crop cultivation, which is still true today, cattle farming being the main resource in this inland region. On the other hand, data recorded for the three Medieval population samples underlined the higher incidence of caries in the Marsan samples than that in the Saint-Michel and Vilarnau samples. In the light of the differences of access to dietary resources among these three population samples [10], we can explain the percentage variation of caries: the Marsan population lived in an inland region in which many silos for cereal crops have been discovered at archaeological sites, so the people's daily food was based on cooked cereal, and cervical carious lesions gave evidence of cariogenic food intake. In Vilarnau, a coastal area, people had Mediterranean food resources that included more fruits, green vegetables, and fish and less carbohydrate than in Marsan. At the Saint-Michel archaeological site, a Medieval suburb of Toulouse in which poor people lived, who had difficulty in obtaining food, the food restriction that affected the population sample can be associated with the lower incidence of caries than that in Marsan. A survey conducted during the Second World War, in a population subjected to dietary restrictions, also shows a decrease in caries frequency [1]. Moreover, Garcin's comparative study [3], concerning medieval juveniles in four European countries, corresponds to our results regarding the geographical site and dietary resources: both coastal and, to a lesser extent, urban sample populations showed a lower dental caries frequency than the inland population, a result suggesting that the coastal population, who ate fish and few carbohydrates, had a less cariogenic diet.

**Figure 2:**
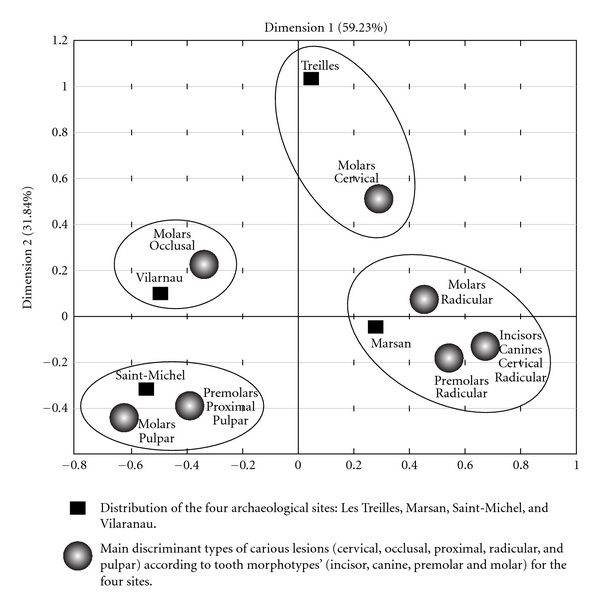
Distribution of the four populations according to caries by principal analysis. In relation to dimension1, Marsan was opposed to Vilarnau and Saint-Michel. In relation to dimension 2, the 3 sites Marsan, Vilarnau, and Saint-Michel were opposed to Les Treilles.

In Figure 2, the Principal Component Analysis (PCA) diagram displays the carious lesion distribution of the four population samples studied in relation to the tooth morphotype and type of carious lesion. Dimensions 1 and 2 include, respectively, 59.23% and 31.84% of information taking account of the five types of caries. We can see that the Chalcolithic sample from Les Treilles is characterized by cervical caries on molars and differs from the three Medieval population samples' distribution. The Marsan sample is related to pulpar and radicular caries on molars, premolars, canines, and incisors, thatis affected by the most severe type of caries. The Saint-Michel and Vilarnau samples show a low incidence of proximal and pulpar caries and occlusal caries on molars.

These French data regarding the comparison and evolution of the carious lesion processes in population samples belonging to two historical periods make an additional contribution and are a part of the history of dental caries. Our results also verify the hypothesis made for the effect of environment and lifestyle, including food preparation, on dental heath.

Our findings are supported by works considering the area of dental caries history from the Chalcolithic to the present time, which most often show an evident relationship between access to food resources, social level, and caries incidence.

Studies considering archaeological human remains usually distinguish two main populations over time: the Hunter-Gatherers, with a very low percentage of dental caries, and the Farmers, whose carious lesion incidence is increased relative to the first group. So, the Chalcolithic Period, when diet was above all rich in hard fibrous vegetables and low in starch and sugars, was characterized by a low caries incidence. For instance, caries frequency varied widely from none to 25% of the teeth for populations of the Metal Age and the Islamic period in the Arabian Gulf [16]. This author points out differences between coastal and inland dwellers on the one hand and, on the other hand, a relationship between little evidence of calculus and low caries frequency and heavy calculus accumulation with high caries frequency because a fibrous diet, poor in carbohydrates, removes calculus deposits, unlike the soft, cooked carbohydrates which cannot have this effect. In all cases, premolars and molars were the teeth most often affected by the carious process. For Eshed et al. [17], changes in food-preparation techniques and nondietary use of teeth can explain the dental health differences before and after the agricultural revolution.

In South America, Cucina et al.'s study [18] analysed patterns of carious lesions occurring in the coastal Maya population of Xcambó in northern Yucatan in the Classical Period. To do this, the study investigated caries in the permanent dentition of adults from the Early (250–550 AD) and Late (550–750 AD) Classical Periods. The results indicate an increase in caries from between 7.4% and 21.2% in the Early Classical to a mean of around 20% in the Late Classical period, but the author stresses on the limitations imposed by interpreting carious lesions solely in terms of single dietary components, such as maize consumption, without taking broader aspects of cultural and socioeconomic relevance into account.

Following the period chronology, works studying population samples who lived over the early centuries AD noted variations in caries incidence related to access to dietary resources and socioeconomic level, as around the Roman historical period [19].

Following the historic chronology, in the Middle Ages, caries incidence increased and affected around 20% of teeth in populations whose food was cooked and included carbohydrates [20, 21].

Later, in the 18th C., Whittaker and Molleson in England [22] drew a parallel between the increase of caries incidence and the increase in the importation of sugar.

Today, works on caries incidence report the impact of food resources on the risk of caries development [23–28]. For instance, during the First and Second Wars, the context of deprivation was related to a steady decline of caries incidence [1, 29]. Moreover, recent studies in the different continents establish relationships between dental health and financial insecurity with low family income and poorly educated parents [24, 26–28]. In this context, very young children, less than one year old [28], are more often affected by caries because of frequent intake of sweet food and drink [23, 25–27] whereas, in Caglar's Byzantine population, the caries prevalence in primary dentition was 0% [20].

These changes in daily eating and dietary models also have consequences on health and quality of life.

To conclude, the population samples studied, over the historical period considered, displayed caries frequency differences related to the geographical area and access to dietary resources. Investigations spread over time and place support our observations and contribute to the idea of a continuing relationship between way of life, food quality and access, socioeconomic level, and dental caries evolution, a reliable predictor of the context of life. Bearing this in mind, the direct, continuous impact of starch and sugar consumption on caries incidence is visible from the Chalcolithic Age to the present time, around the world and across civilizations.

## Figures and Tables

**Table 1:** State of dental arches.

Number of maxillae and teeth	Maxillae	Mandibles	Teeth expected	Teeth present	Teeth lost	
					Antemortem	Postmortem
Les Treilles	0	24	384	157 (40.8%)	45 (11.71%)	182 (47.3%)
Marsan	33	33	1056	791 (74.9%)	56 (5.3%)	140 (13.2)
Saint-Michel	41	41	1312	992 (75.6%)	47 (3.6%)	114 (8.6%)
Vilarnau	58	58	1856	1376 (74.1%)	146 (7.8%)	294 (15.8%)

Total	103	127	4608	3316 (72.0%)	294 (6.3%)	730 (15.8%)

**Table 2:** Number and frequency of tooth presence and absence and caries location for four burial sites.

Burial site	Saint-Michel				Marsan				Les Treilles				Vilarnau			
Tooth type	Molars	Premolars	Canines	Incisors	Molars	Premolars	Canines	Incisors	Molars	Premolars	Canines	Incisors	Molars	Premolars	Canines	Incisors
*Presence/absence*																
Number of teeth expected	492	328	164	328	396	264	132	264	144	96	48	96	696	464	232	464
Teeth present Number	348	281	144	293	293	253	119	126	89	33	21	14	410	401	205	360
Frequency	0.70	0.85	0.87	0.89	0.73	0.95	0.90	0.47	0.61	0.34	0.43	0.14	0.58	0.86	0.88	0.77
Antemortem tooth loss Number	33	7	1	41	41	12	2	1	29	5	3	8	125	15	0	6
Frequency	0.06	0.02	0.06	0.12	0.10	0.04	0.01	0.003	0.20	0.05	0.06	0.08	0.17	0.03	0	0.01
Postmortem tooth loss Number	24	20	8	8	8	13	16	103	26	58	24	74	121	48	27	98
Frequency	0.04	0.06	0.04	0.02	0.02	0.04	0.12	0.39	0.18	0.60	0.5	0.77	0.17	0.10	0.11	0.21

*Caries location*																
Cervical Number	3	0	0	0	15	12	3	3	11	1	0	0	2	1	0	0
Frequency	0.86	0.00	0.00	0.00	5.12	4.74	2.52	2.38	7.28	2.44	0.00	0.00	0.44	0.25	0.00	0.00
Occlusal Number	25	0	0	0	83	30	7	6	5	0	0	0	100	8	0	1
Frequency	7.18	0.00	0.00	0.00	28.33	11.86	5.88	4.76	3.31	0.00	0.00	0.00	22.22	2.00	0.00	0.30
Proximal Number	18	18	2	3	74	43	10	8	2	0	0	0	23	35	8	11
Frequency	5.17	6.41	1.39	1.37	25.26	17.00	8.40	6.35	1.32	0.00	0.00	0.00	5.11	8.73	3.88	3.25
Radicular Number	5	3	0	0	26	22	6	3	2	0	0	0	8	2	0	0
Frequency	1.44	1.07	0.00	0.00	8.87	8.70	5.04	2.38	1.32	0.00	0.00	0.00	1.78	0.50	0.00	0.00
Pulpar Number	28	10	2	1	48	25	7	4	0	0	0	0	28	17	3	3
Frequency	8.05	3.56	1.39	0.46	16.38	9.88	5.88	3.17	0.00	0.00	0.00	0.00	6.22	4.24	1.46	0.89
